# Canine Gastric Cancer: Current Treatment Approaches

**DOI:** 10.3390/vetsci9080383

**Published:** 2022-07-26

**Authors:** Diana Araújo, Inês Cabral, Nuno Vale, Irina Amorim

**Affiliations:** 1 Institute of Biomedical Sciences Abel Salazar (ICBAS), Universidade do Porto (UP), Rua de Jorge Viterbo Ferreira 228, 4050-313 Porto, Portugal; up201809297@up.pt; 2 Fitzpatrick Referrals Oncology and Soft Tissue, 70 Priestley Rd, Guildford GU2 7AJ, UK; ipereiracabral@fitzpatrickreferrals.co.uk; 3 OncoPharma Research Group, Center for Health Technology and Services Research (CINTESIS), Faculty of Medicine, University of Porto, Rua Doutor Plácido da Costa, 4200-450 Porto, Portugal; nunovale@med.up.pt; 4 Department of Community Medicine, Information and Health Decision Sciences (MEDCIDS), Faculty of Medicine, University of Porto, Rua Doutor Plácido da Costa, 4200-450 Porto, Portugal; 5 Associate Laboratory RISE—Health Research Network, Faculty of Medicine, University of Porto, Alameda Professor Hernâni Monteiro, 4200-319 Porto, Portugal; 6 Institute for Research and Innovation in Health (i3S), Universidade do Porto (UP), Rua Alfredo Allen 208, 4200-135 Porto, Portugal; 7 Institute of Molecular Pathology and Immunology (IPATIMUP), University of Porto, Rua Júlio Amaral de Carvalho 45, 4200-135 Porto, Portugal

**Keywords:** human gastric cancer, canine gastric cancer, anticancer drugs, resistance, chemotherapy

## Abstract

**Simple Summary:**

Human gastric cancer is a prevalent cancer worldwide with a high mortality rate. Although sharing many other features, the incidence of gastric cancer is lower in dogs than in humans. Surgery is the first-line treatment; however, it is associated with several complications. Nevertheless, chemotherapy to treat canine gastric cancer has not received much attention, probably due to its late diagnosis, fast progression, low median survival time, and very high mortality rate, along with the lack of publications with concrete scientific results. In this review, we explore the pharmacological approach used in treatment of this often-fatal disease.

**Abstract:**

Human gastric cancer (GC) ranks as the fifth most prevalent cancer worldwide, and is the third leading cause of cancer-related death. The incidence of GC is lower in dogs than in humans, accounting for less than 1% of all canine malignancies. In recent years, efforts have been made to understand the pathogenesis of GC and in find an appropriate therapy to maximize curative results, such as adjuvant chemotherapy treatments in addition to surgery. Although surgery is the first-line treatment, it is associated with several complications. In terms of chemotherapeutic intervention, canine gastric cancer has not received much attention, probably due to its late diagnosis, fast progression, low median survival time, and very high mortality rate, along with the lack of publications with concrete scientific results. In this review, we explore canine GC and the pharmacological approach used in the treatment of this often-fatal disease.

## 1. Introduction

Human gastric cancer (GC) ranks as the fifth most prevalent cancer worldwide, and is the third most common cause of cancer-related death [1]. The incidence of GC is lower in dogs than in humans, accounting for less than 1% of all canine malignancies [2,3,4,5,6,7]. Nevertheless, the prevalence of canine GC might be underestimated, as this disease is commonly diagnosed in older animals and in advanced stages, often causing reluctance by owners to pursue further diagnostic examinations [5]. 

In recent years, progress has been made in understanding the pathogenesis of GC, and efforts have been carried out to find appropriate therapies that could maximize curative results, such as adjuvant chemotherapy treatments in addition to surgical removal alone [8]. 

In human medicine, there are factors that influence treatment, especially when the tumor is advanced, recurrent, metastatic, or even inoperable. In those cases, in order to promote a high median survival time and provide lifetime quality, chemotherapy is highly recommended as an adjuvant therapy (following the surgical removal) or neoadjuvant therapy (prior to other treatment modalities) [9,10]. Depending on the tumor type and of the disease stage, radiation therapy can also be performed [9,10]. 

In veterinary literature, limited information is available regarding the clinical outcome of GC after surgical resection, as well as chemotherapy’s role in the progression of this disease. Indeed, studies evaluating dogs that have had surgery to treat gastric carcinoma have been restricted to case reports and case series that included multiple gastric tumor types [11]. 

In this review, canine GC and the different approaches used in the treatment of this disease, often fatal or associated with a poor prognosis, are thoroughly described. 

## 2. Epidemiology and Risk Factors

The etiology of human GC is complex and not completely understood, with hereditary cancer syndromes, known risk factors, other gastric diseases, and *Helicobacter pylori* infection playing an important role [1]. In dogs, the etiology of GC is unknown, although associations between the development of canine gastric neoplasia and long-term nitrosamine administration, as well as genetic factors such as breed predisposition, have been described [12]. Several studies reported a breed predisposition in Belgian shepherd dogs (Tervuren and Groenendael) [3,5,6,7,13,14], rough collies [5,6,7,14,15], Staffordshire terriers [7,14,15], Chow Chows [6,16,17], and standard poodles [5]. Males present higher incidence of GC, and older dogs, ranging from 7 to 11 years, are most often affected [2,3,5,13,18].

## 3. Diagnosis and Prognosis 

As in humans, clinical signs of GC in dogs are usually mild to absent during the early stages of the disease, with the most common being vomiting, anorexia, weight loss, and lethargy [1,19,20]. The duration of symptoms can range from weeks to many months [19].

About 90–95% of GC in humans are adenocarcinomas, which originate from the epithelial cells of the gastric mucosa. Similarly, adenocarcinoma is also considered the most common neoplastic entity in the stomach of dogs, comprising 50–90% of all canine gastric malignancies [2,4,21].

A preliminary diagnosis is usually obtained using ultrasound or endoscopic examination of the stomach, allowing the mucosa visualization and the collection of biopsies in order to enable a definite diagnosis [5]. Due to the advanced stage of GC at the time of diagnosis and the high frequency of metastasis, early detection remains very important for a successful treatment [5,6]. 

**Figure 1:**
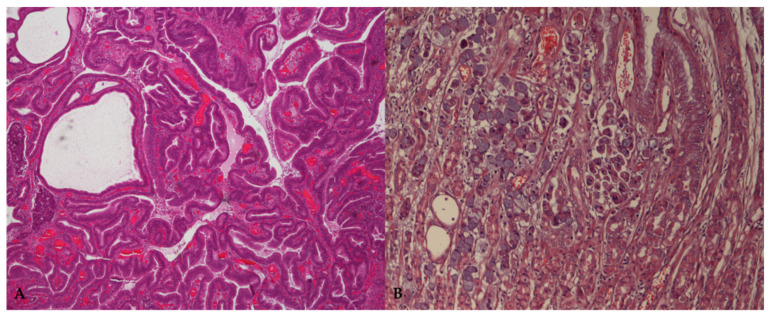
Representative microphotographs of the main histological variants of canine gastric carcinoma. (**A**) Tubulopapillary or intestinal type, according to the WHO and Lauren classification, respectively. HE = 40×. (**B**) Signet ring cell carcinoma or diffuse type, according to the WHO and Lauren classification, respectively. HE = 100×.

Microscopically, according with the WHO classification for domestic animals [22], carcinomas are categorized into papillary, tubular, mucinous, and signet ring cell subtypes, based on the main histological and cytological features of the lesion. In addition, Lauren’s criteria for humans have been successfully adapted to canine GC, placing carcinomas into two major histological subtypes, namely the intestinal and diffuse types [23] (Figure 1).

The prognosis of GC cases is poor, and 70–90% have metastasized by the time of diagnosis or euthanasia [5,6,7,14]. Canine GC is frequently located in the lesser curvature or pylorus, often spreading to other sites such as gastric lymph nodes, omentum, liver, duodenum, pancreas, spleen, oesophagus, adrenal glands, and lungs [3,4,6,7,13]. Indeed, Swann and Holt reported that, in 6 out of 21 cases of dogs with GC, metastasis was found at surgery, with each patient euthanized at this precise moment at the owner’s request [4].

Reported survival times in untreated dogs are less than 3 months after the onset of clinical signs [11]. 

**Figure 2:**
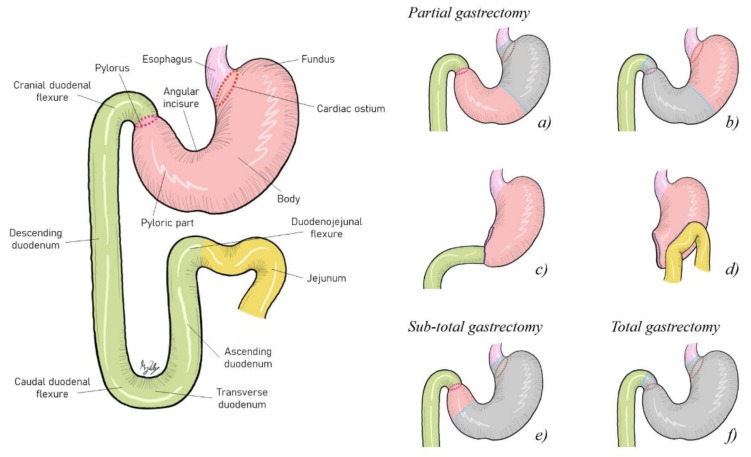
(**a**,**b**) Partial gastrectomy (≤70% of the stomach removed). (**c**) Billroth I (gastroduodenostomy), a reconstruction technique after partial gastrectomy. (**d**) Billroth II (gastrojejunostomy), a reconstruction technique after partial gastrectomy. (**e**) Subtotal gastrectomy (>70% but not complete stomach removal). (**f**): Total gastrectomy (complete stomach removal).

Surgical treatment of gastric neoplasia consists of pylorectomy and gastroduodenostomy (Billroth I) [6,7,24], allowing a wide excision of abnormal pyloric tissue and improving gastric outflow (Figure 2). However, several studies identified postoperative complications. Dogs with gastric neoplasia were not more likely to die due to postoperative complications, but long-term survival was poor, with an overall median survival of 33 days [24].

## 4. Treatment Strategies

### 4.1. Surgical Resection Approach

Several studies described putative complications associated with the surgery aiming to treat canine GC and their association with the overall animal survival (Table 1). Thirteen dogs with different malignant neoplasias and two dogs with benign tumors underwent a pylorectomy and gastroduodenostomy. Eight dogs had complete excision of the neoplastic disease with an overall median survival time of 578 days, and five dogs had incomplete resections of the neoplasia, with an overall survival time of 33 days. In the other two dogs, the excised tissue margins were not reported. Only one patient with incomplete margins resected died within the 14 days of the postoperative period. The overall median survival time of dogs with malignant neoplasia or with metastatic disease was 33 days, which was strongly different from the overall median survival time of patients with benign diseases or without metastatic disease, which was more than 1659 days. Of the eight patients with metastasis, only two died within the 14 days of the postoperative period [24]. 

A study assessed perioperative (intraoperative and/or postoperative) complications in dogs that underwent surgical resection for gastric adenocarcinoma. Ten out of the total number of patients were subjected to partial gastrectomy (≤70% of the stomach removed). Of these: (a) two dogs displayed major intraoperative and postoperative complications, such as spillage of gastric contents and septic peritonitis, surviving only 1 day; and the other had an inadvertent stab incision into the small bowel and septic peritonitis, surviving 190 days; (b) three patients had postoperative major complications, such as cardiopulmonary arrest (surviving 2 days); septic peritonitis (surviving 2 days); and gastric stasis, pancreatitis, and cardiopulmonary arrest (surviving 15 days); (c) three dogs presented intraoperative complications with minor hemorrhage, surviving 2, 3, and 177 days, respectively; (d) two dogs had minor postoperative complications—hypertension was verified in one animal that achieved 274 days of survival time, and the other exhibited hyporexia, surviving 132 days [11]. Furthermore, partial distal gastrectomy and gastroduodenal anastomosis were performed in 10 dogs with GC, and the generic postoperative complications consisted of discomfort, vomiting, and diarrhea for 10–15 days. The overall median survival time reached was 72 days. Two patients lived for 3 and 4 years, and the causes of their deaths were unrelated to the gastric tumor; six dogs were euthanized due to disease progression, and the survival time ranged from 30 days to 20 months; one dog died 3 days after surgery due to unrelated problems; and another patient lived at least 5 months [14]. In another investigation, a dog with GC was euthanized 7 days after partial gastrectomy due to postoperative detection of pericardial effusion. In a dog that had gastric dilatation-volvulus in addition to gastric adenocarcinoma, a partial gastrectomy and splenectomy were executed. However, the patient was euthanized 2 days after surgery due to disseminated intravascular coagulation and ventricular arrhythmias [12].

Five out of the total patients underwent the Billroth I surgical procedure (gastroduodenostomy, a reconstruction technique after partial gastrectomy). Among these: (a) one patient had pancreatitis as a major postoperative complication, surviving 16 days; (b) another suffered hypertension as a minor intraoperative complication, as well as ascending cholangiohepatitis and pancreatitis as postoperative complications, and had a survival time of 71 days; (c) another animal presented minor intraoperative complications, such as a major hemorrhage and a 2nd-degree atrioventricular block, and had a survival time of 49 days; (d) the last two dogs exhibited major postoperative complications—one presented severe pancreatitis and intermittent hypoglycemia and survived 258 days, while the other pulled out the gastrostomy tube, but survived 183 days [11]. Four patients received a Billroth I, and one dog was euthanized 3 days after surgery due to persistent vomiting, while another two dogs were euthanized after 6 weeks, and the other one after 10 months due to vomiting and anorexia. In two cases, gastrojejunostomy (Billroth II) was performed due to an extension of the neoplasia, and the patients were euthanized 4 and 5 weeks after surgery as a consequence of recurrence vomiting and anorexia [4]. 

In another two patients that were subjected to a subtotal gastrectomy (>70% but not complete stomach removal): one displayed a major intraoperative complication that lead to a subtotal gastrectomy and postoperative complications such as septic peritonitis, surviving 13 days; the second patient presented a minor intraoperative hemorrhage and postoperative complications such as vomiting, accomplishing a survival time of 93 days [11]. 

Another canine patient with gastric adenocarcinoma that suffered total gastrectomy was euthanized after 240 days due to discomfort exhibited during and after eating [25]. 

### 4.2. Pharmacological Approach

Veterinary oncology is an expanding area, and chemotherapy protocols have been established in common canine neoplasms. The most common treatment of canine GC is wide surgical resection in order to obtain clean margins. However, due to nonspecific clinical signs, the disease is often identified in an advanced stage, and its extension and location usually precludes a surgical approach [1,11,19,20]. 

Although still unclear, chemotherapy protocols used in various studies include single agents or combination therapies employed after surgical resection (see Table 2 and Supplementary Materials) [4,11,12,26,27]. 

In terms of chemotherapeutic intervention, canine GC has not received much attention, probably due to its rare occurrence, late diagnosis, fast progression, low median survival time, and very high mortality rate, along with a lack of publications with concrete scientific results.

#### 4.2.1. Chemotherapy Schemes

##### Carboplatin and Cisplatin

Platinum drugs are an important class of antitumor compounds. Alone or in combination with other agents, cis-diamminedichloroplatinum (II) (cisplatin) and its analogues display a significant impact on the treatment of various solid tumors. However, the high toxicity profile observed with cisplatin has led to the development of platinum analogues that are more tolerable and active against different tumor types, including some that are resistant to cisplatin [28]. 

In addition to cisplatin, two other platinum complexes are currently approved for use: cis-diamminecyclobutanedicarboxylato platinum (II) (carboplatin) and 1,2-diaminocyclohexaneoxalato platinum (II) (oxaliplatin) [28]. The antitumor activity of these agents is through their covalent binding to DNA, causing bifunctional lesions and inter- or intrastrand cross-links [10,29,30,31]. 

Carboplatin is a second-generation platinum-compound chemotherapeutic agent that can be safely used in companion animals. It was developed for humans in an attempt to minimize the numerous side effects of cisplatin, including nephrotoxicity, nausea, vomiting, neurotoxicity, ototoxicity, and myelosuppression, maintaining comparable tumor cytotoxicity [10,30,31,32,33]. The ammine carrier ligands are the same in both the carboplatin and cisplatin molecules [28]. Carboplatin differs from cisplatin in its cyclobutanedicarboxylate group at the position of the two chlorides in cisplatin [28,34], resulting in a complex with reduced renal toxicity [28]. Cisplatin is considered the most active antineoplastic agent in animal systems, and some veterinary oncologists consider it more potent than other platinum agents [33,35], as it used both in human and veterinary medicine. However, cisplatin is not indicated for cats, as it is highly toxic to them [10,29,30]. Due to cisplatin’s high nephrotoxicity, carboplatin is the most commonly used platinum analogue, being a more cost-effective substitute to cisplatin [31,33]. However, special attention should be paid to renal patients, since carboplatin is excreted in the urine and presents some nephrotoxicity, although less than that of cisplatin [10,29,30]. In veterinary oncology, carboplatin is used in the treatment of osteosarcoma and in a diversity of sarcomas and carcinomas, such as intestinal and prostate carcinomas and anal sac adenocarcinoma. The toxicities related to these therapies consist of nausea/vomiting and myelosuppression, in addition to nephrotoxicity [10,29,30,31]. Generally, in dogs that weigh less than 15 kg, a dose of 300 mg/m^2^ of carboplatin is administered intravenously (IV) every 3–4 weeks, while 350 mg/m^2^ is administered in dogs larger than 15 kg [30,31].

Carboplatin alone was used in some studies aiming the treatment of canine GC, although different protocols were followed: (a) 250 mg/m^2^ carboplatin for the first dose and 200 mg/m^2^ for the subsequent treatments, three times in a period of 10 weeks. Despite the fact that the lesion inspired a poor prognosis, the status of the patient was well controlled after treatment achieving 30 months of survival after surgery [26]; (b) 280 mg/m^2^ IV every 3 weeks, being administered five of the six intended doses. The survival time after surgery was 272 days, with the animal presenting grade I lethargy, vomiting, diarrhea, and grade II neutropenia [11]; (c) two cases received the same protocol: four doses of 300 mg/m^2^ IV every 3 weeks, and the survival times were 93 days with grade I neutropenia after the 3rd dose and 383 days with grade I diarrhea (the animal was still alive at the end of the study), respectively [11]. 

##### Doxorubicin

Antitumor antibiotics are natural products that originate from microbial fermentation, including anthracyclines (doxorubicin) and a synthetic analogue of the anthracenediones (mitoxantrone), mitomycins, and actinomycins, which have produced clinically useful compounds with various mechanisms of action. Doxorubicin (DOX) reacts with a variety of cellular components, and its activities include DNA intercalation and inhibition of RNA and DNA polymerases and topoisomerase II, alkylation of DNA, reactive oxygen generation, perturbation of cellular Ca^2+^ homeostasis, inhibition of thioredoxin reductase, and interaction with plasma membrane components. DOX is administered intravenously and is extensively distributed to all tissues [10,29,30,31]. 

The dose-limiting toxicities associated with DOX therapy are infusion-rate-dependent hypersensitivity, myelosuppression, gastrointestinal toxicity, and a cumulative dose-related cardiotoxicity [10,29,30,31]. In dogs, in order to limit DOX’s potential toxicity, some protocols optimized the DOX cumulative dose to 180–240 mg/m^2^ after a formal cardiac evaluation [29]. Dexrazoxane can be administered with DOX to reduce cardiac toxicity [10,29,30,31]. DOX must be carefully administered, since it is a vesicant drug and its extravasations can cause tissue necrosis, which may require further surgical intervention [9,10,29,31]. DOX is the most active single agent available for different types of tumors in companion animals. This drug can be used alone or in combination protocols for several malignancies [10,29,31]. 

In dogs, the conventional dosing administration is 30 mg/m^2^ every 3 weeks for dogs larger than 15 kg, and 1 mg/kg for smaller patients [10,29,30,31]. Since a universal prescription protocol has not been established yet, dose adjustments should be anticipated following the initial DOX administration. It is important to evaluate cardiac performance through echocardiography and electrocardiography prior to each DOX administration to detect any murmurs, arrhythmias, or pulse deficits [29,30,31], especially in patients with cardiac disease or breeds with dilated cardiomyopathy predisposition [30,31].

Doxorubicin alone was used in four cases of canine gastric adenocarcinoma; in all cases, 30 mg/m^2^ IV was administered, but with a different frequency for each. One dog began systemic chemotherapy with DOX on day 43 after surgery, and it was repeated on days 69, 90, and 111. The patient responded well to therapy initially; however, after day 90 (3rd DOX dose), the dog began to lose weight, and on day 114, it presented extreme lethargy, fever, increased respiratory effort, and recurrent pneumonia, and was euthanized [27]. Another dog with adenocarcinoma received five doses of DOX postoperatively, and the survival time was 81 days [12]. In another study, a dog with grade II anorexia was treated with four doses of adjuvant DOX every 3 weeks, and survived 177 days [11]. More recently, a canine patient with gastric carcinoma was treated with surgery (Billroth I) and then was subjected to six DOX administrations, the specific doses of which were not specified, and survived 1 year [36].

**Table 2:** Chemotherapy protocols used for canine gastric cancer.

Adjuvant Therapy	Dose and Frequency	Surgical Removal	Survival Time	Histology	Reference
Carboplatin	250 mg/m^2^ carboplatin for the first injection and 200 mg/m^2^ for the 3 subsequent treatments, 4 times for 13 weeks	Yes	30 months	Adenocarcinoma	[26]
	280 mg/m^2^ IV every 3 weeks × 5/6 intended doses	Yes	272 days		[11]
	300 mg/m^2^ IV every 3 weeks × 4/4 doses	Yes	93 days		
	300 mg/m^2^ IV every 3 weeks × 4/4 doses	Yes	383 days **		
Carboplatin/5-FU	Carboplatin: 275 mg/m^2^ IV, 1 dose single agent Carboplatin/5-FU: 200 mg/m^2^/150 mg/m^2^ IV every 3 weeks × 5/5 doses	Yes	553 days **		
	5-FU: 150 mg/m^2^ IV as a slow push Carboplatin: 200 mg/m^2^ IV over 10 min, 1 h after 5-FU	-	79 days	Metastatic GC	[37]
		-	26 days		
Carboplatin Toceranib *	Carboplatin: 285 mg/m^2^ IV every 3 weeks × 6/6 doses Toceranib: 2.5 mg/kg MWF × 3 months	Yes	354 days	Adenocarcinoma	[11]
Carboplatin Mitoxantrone *	Carboplatin: 240 mg/m^2^ IV × 1 dose Mitoxantrone: 5 mg/m^2^ IV every 3 weeks × 6/6 doses	Yes	190 days		
Gemcitabine/Carboplatin Toceranib *	Week 1—gemcitabine/carboplatin: 57 mg/m^2^/285 mg/m^2^ IV Week 2—gemcitabine: 57 mg/m^2^ IV × 4/4 cycles Toceranib: 3.3 mg/kg PO MWF	Yes	564 days		
Carboplatin Cyclophosphamide Doxorubicin	Carboplatin: 300 mg/m^2^ every 3 weeks × 4/4 doses Cyclophosphamide: 15 mg/m^2^ daily × 2 months Doxorubicin: dose not reported, every 3 weeks × 3 doses	Yes	274 days		
Toceranib Cyclophosphamide	Toceranib: 1.7 mg/kg PO MWF Cyclophosphamide: 12.5 mg/m^2^ PO every other day	Yes	1902 days		
Doxorubicin	30 mg/m^2^ IV on day 43 following surgery and repeated on days 69, 90, and 111	Yes	114 days		[27]
	30 mg/m^2^ IV 5 doses	Yes	81 days		[12]
	30 mg/m^2^ IV every 3 weeks × 4/4 doses	Yes	177 days	Adenocarcinoma	[11]
	6 treatments	Yes	1 year	Carcinoma	[36]
Doxorubicin Cyclophosphamide	Week 1—doxorubicin: 27 mg/m^2^ IV Weeks 2 and 3—cyclophosphamide: 222 mg/m^2^ PO divided over 2 days × 2/5 intended cycles	Yes	101 days	Adenocarcinoma	[11]
	Doxorubicin: 25 mg/m^2^ IV Cyclophosphamide: 50 mg/m^2^ PO for 4 days	Yes	9 weeks		[4]
Doxorubicin Carboplatin	1 treatment	No	21 days	Carcinoma	[36]
Gemcitabine	222 mg/m^2^ IV × 1 dose	Yes	71 days	Adenocarcinoma	[11]
	675 mg/m^2^ IV every 2 weeks × 4/4 doses	Yes	97 days		
Toceranib	1.7 mg/kg PO MWF	Yes	280 days		
	2.7 mg/kg PO × 2 doses	Yes	403 days		
	3 mg/kg PO MWF	Yes	135 days		
	1.5 mg/kg PO × 2 doses	Yes	49 days		
	3.4 mg/kg PO MWF	Yes	132 days **		
Mitoxantrone	5.5 mg/m^2^ diluted 1:1 in 0.9% NaCl, then again in 1 mL/4.5 kg, intracavitary	Yes	311 days		
5- FU/Cyclophosphamide	5-FU: 150 mg/m^2^ IV Cyclophosphamide: 50 mg/m^2^ PO for 4 days every 2 weeks for two cycles	No	9 weeks	Adenocarcinoma	[4]
FAC protocol Cis-platinum	FAC protocol: doxorubicin, 25 mg/m^2^ IV, and cyclophosphamide, 75 mg/m^2^ PO for 4 days on week 1; 5-FU, 150 mg/m^2^ IV on weeks 2 and 3 for 8 cycles Cis-platinum: 60 mg/m^2^ IV over 6 h, every 3 weeks for two cycles	No	7.5 months		
Prednisolone	0.5–1.0 mg/kg/day	Yes	104 days **		[38]
Piroxicam	0.3 mg/kg/day	No	374 days		
Piroxicam Cyclophosphamide	Piroxicam: 0.3 mg/kg/day Cyclophosphamide: 15.0 mg/m^2^/day	Yes	1366 days		
Piroxicam Prednisolone	Piroxicam: 0.3 mg/kg/day Prednisolone: 0.5–1.0 mg/kg/day	Yes	1250 days **		

* Started after cytotoxic chemotherapy; ** alive at the end of the study; MWF: Monday/Wednesday/Friday; IV: intravenous; 5-FU: 5-fluorouracil; PO: per os.

##### Mitoxantrone

Mitoxantrone is a synthetic DOX analogue that has the same activity of DOX in DNA intercalation and inhibition of RNA and DNA polymerases and topoisomerase II [9,10,29,30,31]. However, mitoxantrone does not cause oxidative damage to cells, and is less likely to generate reactive oxygen species. 

This drug is extensively distributed to tissues, and minimal levels are long-lasting after IV administration. A portion of mitoxantrone is not metabolized (<30%) and is excreted unchanged in the urine and feces [9,10,29,31]. Dose-limiting toxicities consist of gastrointestinal disorders, myelosuppression, and perivascular damage with extravasation [9,10,29,30]. Cardiotoxicity is rare in humans and has not been reported in dogs [29,30,31]. 

In dogs, mitoxantrone (5 to 6 mg/m^2^ IV slow bolus every 3 weeks) is used as a cardiac-sparing anthracycline when the patient reaches the cumulative level of DOX or there is evidence of cardiomyopathy and the patient is at risk of damage with DOX administration [9,29,30,31]. 

Intracavitary mitoxantrone (5.5 mg/m^2^ diluted 1:1 in 0.9% NaCl, then again in 1 mL/4.5 kg) was used in a dog with gastric adenocarcinoma as a palliative intracavitary chemotherapy to treat metastatic bicavitary effusion. It was well tolerated and provided a survival time of 311 days after surgery [11]. 

##### Gemcitabine

Antimetabolites consist of agents that inhibit the use of cellular metabolites during cell growth and division processes. These agents are analogues of compounds used in normal metabolism and in anabolic processes associated with DNA replication in cancer chemotherapeutics [10,29]. 

Gemcitabine (2′,2′-difluorodeoxycytidine) is an analogue of difluorinated deoxycytidine that has an important clinical activity against numerous human solid tumors and hematologic malignancies [10,29,31,39]. Gemcitabine is actively transported into cells by nucleoside transporters, requiring human equilibrative nucleoside transporter 1 (hENT1) action [29,39]. This drug requires intracellular activation to exhibit its cytotoxic effects, as it is activated to the triphosphate metabolite active form by the same enzymatic machinery [39]. This is incorporated into DNA, resulting in chain termination and inhibition of DNA synthesis and function [10,39].

This drug is administered through an IV route, being relatively well tolerated when used as a single agent. The main dose-limiting toxicity consists of myelosuppression, but in the presence of other antimetabolites, gemcitabine produces greater hematologic toxicity in longer infusions [10,39]. In higher doses, gastrointestinal toxicity can be moderate to significant [10,29]. 

In veterinary oncology, gemcitabine has been rarely reported, although some studies used it as a single agent or in drug combinations, or combined it with radiation treatment. In dogs, dose regimens involve both high-dose (800 mg/m^2^ IV over 20 to 30 min, every week for 4 weeks) or low-dose (25 to 50 mg/m^2^ IV, once or twice a week per protocol) options, depending on the use of other cytotoxics [29,31]. 

In one study, two dogs with gastric adenocarcinoma received one dose of 222 mg/m^2^ IV and four doses of 675 mg/m^2^ IV of gemcitabine every 2 weeks after surgery, which was well tolerated without complications or only with grade 1 lethargy, resulting in 71 days and 97 days of survival time, respectively [11].

##### 5-Fluororacil

5-Fluororacil (5-FU) is a halogenated analogue of uracil that enters cells via the facilitated uracil base transport system and is intracellularly converted to active nucleotide forms via phosphorylase and kinase reactions to yield monophosphate, diphosphate, and triphosphate forms of both fluorouridine and fluorodeoxyuridine, which are incorporated into RNA and DNA and interfere with its synthesis and function [10,29,30,31,39]. This drug is administered via IV and is widely metabolized in several tissues by dihydropyrimidine dehydrogenase to dihydrofluorouracil, and further catabolized to α-fluoro-β-alanine, ammonia, and carbon dioxide; approximately 90% of an administered dose is metabolized [29]. In humans, 5-FU and its analogue compounds are used in the treatment of solid tumors, such as gastrointestinal malignancies. It is also used in combination therapies to treat metastatic colorectal cancer and as an adjuvant therapy for early-stage colon cancer [39]. 

In dogs, 5-FU can be administered topically and intralesionally, and causes a dose-dependent myelosuppression, gastrointestinal toxicity, and neurotoxicity. However, 5-FU is rarely used for management of epithelial tumors (hepatic, pancreatic, renal, mammary), with the protocol for this being 150 mg/m^2^ of the reference dose IV weekly [10,29,30,31]. 

##### Masitinib and Toceranib

In veterinary medicine, there are two kinase inhibitors approved for use in dogs: masitinib and toceranib [40]. Masitinib is a small-molecule, potent, and selective phenylaminothiazole-type tyrosine kinase inhibitor (TKI)that mostly targets the c-Kit receptor and PDGFRα/β [30,40,41]. In dogs with mast cell tumor, masitinib improved time to progression, and better outcome was noted in dogs with mast cell tumors harboring KIT mutations. Dogs treated with long-term masitinib presented benefits in long-term survival and disease control when compared to the patients treated with a placebo [42,43]. 

Toceranib is a tyrosine kinase inhibitor that has antiangiogenic and antitumor properties via the targeting of molecules such as platelet-derived growth factor receptor (PDGFR), vascular endothelial growth factor receptor (VEGFR), KIT protooncogene receptor tyrosine kinase (KIT), FMS-like tyrosine kinase 3 (FLT3), and colony-stimulating factor 1 receptor (CSF1R). Toceranib has demonstrated activity in mast cell tumors, sarcomas, and carcinomas, and has been used in the treatment of various solid tumors [30,31,40].

A study assessed the treatment with toceranib of different types of canine adenocarcinomas (apocrine gland anal sac, small intestinal, lung, and renal cell carcinoma). The results suggested that the effects of toceranib might depend on the type of adenocarcinoma. The dogs tolerated the therapy well, although they presented a higher rate of adverse events when compared to those treated with surgery alone. Postoperative adjuvant therapy with toceranib was associated with a longer time to progression when compared to those treated with surgery alone [44].

In another study, five dogs with gastric adenocarcinoma were treated with toceranib after surgery using different dose regimens and frequencies: (i) 1.7 mg/kg PO on Monday/Wednesday/Friday (MWF); (ii) two doses of 2.7 mg/kg PO MWF; (iii) 3 mg/kg PO on MWF; (iv) two doses of 1.5 mg/kg PO on MWF; and (v) 3.4 mg/kg PO on MWF. In three dogs, this treatment was finished shortly after initiation due to the adverse effects, such as grade II diarrhea and grade I lethargy; in the other two cases, toceranib was well tolerated and was continuously used until disease progression was noted. The survival time of this therapy ranged from 49 to 403 days (280 days, 403 days, 135 days, 49 days, and 132 days, respectively) [11].

##### Cyclophosphamide

Alkylating agents are a group of anticancer drugs whose mechanism of action consists of the covalent binding of alkyl groups to cellular macromolecules; these agents target DNA, in which they form mono- or bifunctional adducts that produce inter- or intrastrand cross-links [29,30,45]. 

Cyclophosphamide is a nitrogen mustard prodrug that involves cytochrome p450 metabolism to release active alkylating species [29,31,45]. Cyclophosphamide is inactive in the absence of metabolic activation, which occurs via microsomal mixed-function oxidases, mainly in the liver [10,29,30,45]. This activation requires ring oxidation to 4-hydroxycyclophosphamide (4-OHCP), spontaneous and reversible ring opening to the amino aldehyde aldophosphamide, and further irreversible breakdown of aldophosphamide to phosphoramide mustard and acrolein [29,30,45].

The alkylating agents are frequently used as single- or multiagent protocols in the treatment of different types of cancer, with cyclophosphamide as the most widely used alkylating agent against a great variety of tumors (lymphoma, leukemia, mammary carcinoma, and sarcomas). The primary dose-limiting toxicity of these agents is bone marrow suppression (neutropenia), with secondary restraining effects in the proliferation of intestinal mucosa cells [10,30,31,45].

In dogs, gastrointestinal toxicity is not common. However, sterile hemorrhagic cystitis and alopecia in breeds with continually growing hair are frequent manifestations of toxicities [10,29,30,31]. Cyclophosphamide can be administered either as a bolus dose (250 mg/m^2^) by PO or IV route in the dog, or used as a metronomic chemotherapy, in which it is administered long-term in a continuous basis at low doses and with minimal pauses [29,30,31]; this has been studied in hemangiosarcoma, soft tissue sarcoma, and transitional cell carcinoma in dogs [9].

##### Prednisolone

Prednisone (17α,21-dihydroxypregna-1,4-diene-3,11,20-trione) and prednisolone (11β,17α,21-trihydroxypregna-1,4-diene-3,20-dione) are synthetic analogues of cortisol. Prednisone is converted to prednisolone in the liver. Both can be used alone or in combination with other immunosuppressive drugs to treat transplant rejection and autoimmune diseases. Prednisolone is administered via IV as a prodrug [30,46,47,48] that induces apoptosis [29]. These drugs are used as chemotherapy agents, usually in combination therapy, in the treatment of acute lymphatic leukemia, chronic lymphatic leukemia, thymoma non-Hodgkin, lymphoma, multiple myeloma, and breast cancer [10,49]. 

In companion animals, prednisolone is used in the treatment of lymphoid neoplasias, mast cell tumors, and brain tumors. This drug is well tolerated in dogs in a short time period. Usually the patients receive 2 mg/kg or 40mg/m^2^ PO daily [29,31]. Prednisolone can also be used to reduce the toxicity profile of chemotherapy, and anti-inflammatory doses can be administered (0.5 to 1.0 mg/kg PO once daily) [29]. 

A patient with gastric adenocarcinoma that suffered mucosal resection of the pylorus was treated with 0.5–1.0 mg/kg/day of prednisolone, and the survival time recorded was at least 104 days without recurrence, as the animal was alive at the end of the study [38].

##### Piroxicam

Some drugs were not conceived to be used as an anticancer therapies, but some of them can inhibit angiogenesis, and therefore exhibit an antineoplastic profile; these include inhibitors of matrix metalloproteinase (MMP) and cyclooxygenase (COX). 

Piroxicam is a nonsteroidal anti-inflammatory drug that belongs to the oxicam family [10,30,31,50]. It is the nonselective COX inhibitor with the longest half-life, and is completely absorbed after oral administration [30,51]. Piroxicam’s side effects consist of gastrointestinal and skin reactions [10,30,51].

The potential of piroxicam as a COX inhibitor in the treatment of angiogenesis has been studied in canine transitional cell carcinoma [10,31,52]. In dogs, the piroxicam dose is 0.3 mg/kg PO once daily [10,30,53]. Although it is a well-tolerated drug, the side effects include gastrointestinal toxicity, such as ulceration and renal toxicity [10,30,31,53,54]. 

A dog with gastric adenocarcinoma was treated with 0.3 mg/kg/day of piroxicam; however, the animal died 374 days later due to tumor progression [38]. 

##### Combination Therapies 

The effectiveness of the combination of different chemotherapy drugs when compared to single-agent therapies is due to the overcoming of the natural and acquired resistance of tumor cells and the use of agents that diverge in dose-limiting adverse effects. In humans, when compared to the single agent, combination chemotherapy has shown a curative potential in some malignancies. In veterinary oncology, combination chemotherapy was successful in the treatment of canine lymphoma [29].

**Figure 3:**
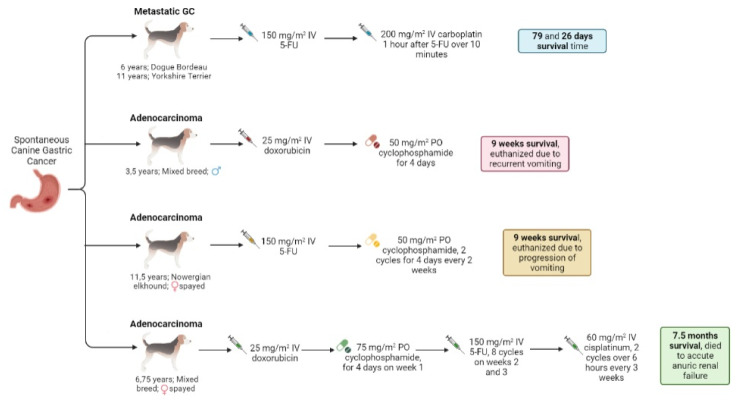
Schematic representation of the most common therapeutical schemes used for canine gastric cancer treatment, based on the available literature [4,37].

Some investigations used combination-chemotherapy protocols as a treatment approach to canine GC (Figure 3). Carboplatin/5-FU was used in gastric adenocarcinoma: a single dose of 275 mg/m^2^ carboplatin IV was administered after surgery, followed by five doses of 200 mg/m^2^ carboplatin plus 150 mg/m^2^ 5-FU IV every 3 weeks, without adverse effects and with a survival time of 553 days, as the was patient alive at the end of the study [11]. Another assay in dogs demonstrated that a combination protocol using 5-FU and carboplatin was well tolerated, and remains a reasonable treatment protocol for various carcinomas, especially gastrointestinal forms [37]. In two cases of canine metastatic gastric carcinoma, a slow IV push of 150 mg/m^2^ 5-FU was administered, and 1 h later, 200 mg/m^2^ IV of carboplatin was added over 10 min, and the survival times were 79 and 26 days, respectively [37]. 5-FU can also be used in canine carcinomas in combination with other platinum agents; in particular, cisplatin and oxaliplatin. However, future studies in a larger cohort of dogs are needed to clearly assess the treatment response and the potential toxicity [37]. Nevertheless, two patients with gastric carcinoma were treated with carboplatin and doxorubicin, and the survival times were no longer than 21 days [36].

In one dog with gastric adenocarcinoma, toceranib administration (2.5 mg/kg on MWF for 3 months) began after cytotoxic chemotherapy of six doses of 285 mg/m^2^ IV carboplatin every 3 weeks, with grade III neutropenia, and the survival time registered was 354 days [11]. In another case, 3.3 mg/kg of toceranib was administered PO on MWF after a cytotoxic chemotherapy effect of 57 mg/m^2^ of gemcitabine plus 285 mg/m^2^ of carboplatin IV on week 1, and four cycles of 57 mg/m^2^ IV of gemcitabine on week 2, with neutropenia and grade II thrombocytopenia, resulting in 564 days of survival time. The same circumstances occurred in a case in which six doses of 5 mg/m^2^ IV mitoxantrone were administered every 3 weeks, and after chemotherapy, one dose of 240 mg/m^2^ IV carboplatin, with a grade I elevation in creatinine. The survival time was 190 days [11].

A multiagent protocol to treat gastric adenocarcinoma used a combination of carboplatin (four doses of 300 mg/m^2^ every 3 weeks), cyclophosphamide (2 months of 15 mg/m^2^ daily), and doxorubicin (three doses every 3 weeks; dose not reported), and the survival time achieved was 274 days without side effects [11].

Cyclophosphamide can be used in combination with different drugs, such as toceranib, doxorubicin, 5-FU, and piroxicam. In order to treat adenocarcinoma, 1.7 mg/kg PO of toceranib was applied on MWF and 2.5 mg/m^2^ of cyclophosphamide PO on the other days, resulting a survival time of 1902 days, with grade II anorexia (toceranib). Two different protocols of doxorubicin and cyclophosphamide were administered in cases of adenocarcinoma: in the first, the patient received 27 mg/m^2^ IV of doxorubicin in week 1, while in weeks 2 and 3, the patient received 222 mg/m^2^ PO of cyclophosphamide divided over 2 days, fulfilling two of the five intended cycles. The animal reached 101 days of survival time without side effects reported [11]; the second protocol used 25 mg/m^2^ IV of doxorubicin and 50 mg/m^2^ PO of cyclophosphamide for 4 days after surgery due to the presence of metastasis. This patient was euthanized 9 weeks after diagnosis due to recurrent vomiting. When used in combination to treat canine gastric adenocarcinoma, 5-FU (150 mg/m^2^ IV)/cyclophosphamide (two cycles of 50 mg/m^2^ PO for 4 days every 2 weeks), the owner elected euthanasia 9 weeks after the diagnosis due to the progression of vomiting [4]. The FAC protocol was used in another dog with gastric adenocarcinoma, consisting of the combination of doxorubicin (25 mg/m^2^ IV), cyclophosphamide (75 mg/m^2^ PO for 4 days in week 1), and 5-FU (8 cycles of 150 mg/m^2^ IV in weeks 2 and 3), plus two cycles of 60 mg/m^2^ IV of cis-platinum over 6 h every 3 weeks. The patient died 7.5 months after diagnosis due to acute anuric renal failure [4]. Another canine patient with gastric adenocarcinoma that was subjected to multiple surgeries was treated with piroxicam (0.3 mg/kg/day) and cyclophosphamide (15 mg/m^2^/day). The survival time was 1366 days, and the cause of death was attributed to gallbladder rupture [38].

Another protocol consisted of endoscopic polypectomy and the administration of 0.3 mg/kg/day of piroxicam and 0.5–1.0 mg/kg/day of prednisolone. The survival time was 1250 days without recurrence, and the dog was alive at the end of the study [38].

#### 4.2.2. Ethical Implications of Chemotherapy

Nowadays, companion animals assume a major role in human families. The balance between the quality and duration of such animals’ lives can be a sensitive matter, but hardly understandable by the owner due to the stronger pet–owner relationship. Therefore, in recent years, an increasing demand has been observed in the search for basic and specialized treatment of animals with cancer [55,56,57]. The human–animal bond has led veterinary oncology to follow and resemble human medicine, and even to some pet owners refusing euthanasia as a tool for suffering relief [56,57]. Frequently, preserving patients’ life quality and extending the lifespan cannot coexist. 

Cancer management in companion animals should begin with a discussion with the pet owner about the potential risks, benefits, toxicity, costs, and time commitment of treatment [27]. Pain encompasses sensory, affective/emotional, and functional elements resulting from a variety of causes, and can last for an unpredictable amount of time. Assessment of pain in dogs can be difficult, and with respect to the gastrointestinal tract, the pain may be difficult to localize, and the animal might exhibit vague signs and behavioral changes [58,59]. Cancer patients can also suffer from pain caused by treatment options such as chemotherapy, radiotherapy, or surgery. The pain incidence related to cancer in animals and the effectiveness of analgesic therapy are difficult to estimate. Nevertheless, recognition and mitigation of pain in those cases is crucial for maintenance of life quality [9,58]. 

Generally, surgery is the best treatment modality if the primary tumor can be completely removed with adequate morbidity. However, some tumors may be difficult to excise due to extensive involvement [9,55]. 

Chemotherapy is used to reduce or prevent tumor spreading, but has a limited therapeutic index and can cause substantial or fatal toxicity, so the negative side effects and the beneficial outcome that this therapy can have on the prognosis and survival time in animal patients should be evaluated [9,60]. In veterinary oncology, errors in dose calculations are more likely to occur, since chemotherapeutic agents can be dosed in milligrams/kilogram (mg/kg) or milligrams per meter squared (mg/m^2^). In addition, breed-specific characteristics can induce side effects [9]. 

A study investigated the factors that may influence owners to use chemotherapy to treat their animals. It suggested that owners’ beliefs, their previous experiences with chemotherapy, and their consciousness of adverse effects over possible benefits may influence their choices in using this treatment in their animals. A reduction in the quality of life was expected during and after the treatment by the majority of the participants. However, most of the owners thought there would be an improvement in the life quality of their animals after chemotherapy, but the survival time after therapy was overestimated, which led to false expectations. Nevertheless, fewer owners would choose this treatment if the extended survival time was less than 3 months, demonstrating that animals’ life quality is important in the decision process. Interestingly, the owners chose chemotherapy treatment based on their perspective, rather than that of their animals. Thus, it is imperative to enlighten owners regarding the chemotherapeutic procedure and its effects by emphasizing the life quality, incidence, and expression of the adverse effects, so they can make rational and informed choices [60].

## 5. Conclusions and Future Perspectives

Survival time is frequently used as the primary end point because it is easy to determine. However, in veterinary oncology, it can be affected by owner-driven factors such as delaying the therapy’s initiation or euthanasia [44]. Survival time can also be influenced by different prognostic factors, such as intraoperative complications and adjuvant chemotherapy administration. In most cases, adjuvant chemotherapy is associated with an improved survival time. However, the available data should be interpreted carefully, since they were mainly the result of retrospective studies, and the treatments analyzed were not randomized. Therefore, the current results could have been influenced by a selection bias that may have existed in the therapy selection or by the timing of the euthanasia. Additionally, this last factor may depend on an owner’s will in pursuing chemotherapy, allowing a longer treatment course before considering euthanasia [11]. Furthermore, recurrence of clinical signs was assumed to be associated with GC progression [4].

Some limitations of these studies included a small sample (due to the rare occurrence of canine GC), a lack of knowledge regarding the disease’s clinical stage, and variations in the surgical procedure protocol, as well as the fact that, in most of the cases in which treatment was attempted, postmortem examinations were not performed [4,11,38]. On the other hand, the efficacy of specific chemotherapeutic protocols could not be analyzed due to the variations in the drugs, dose, duration, and frequency used in each case, making it difficult to deduce a reliably effective chemotherapeutic protocol to treat canine GC. Furthermore, meaningful associations with histologic features and outcome were not assessed due to the limited cases and the inconsistencies and subjectivity of the information provided by both clinicians and pathologists. However, these findings justify additional investigations focusing on the role of chemotherapy in the treatment of canine GC [11]. Future studies must evaluate surgical and histologic margins and the association between histopathologic features and canine GC biologic behavior, as well as explore whether the combination of surgery and adjuvant therapy can lead to an improved outcome in dogs with GC, and most likely should include target therapy in those cases [11,12].

In humans, chemotherapy improves survival, and combination therapies also improve survival when compared to a single-agent treatment. In patients subjected to target therapy with HER-2-positive tumors, protocols using trastuzumab in combination with capecitabine or 5-FU in combination with cisplatin have proven to be valuable. For those patients with tumours that are HER-2 negative, protocols with two- and three-drug combinations, such as those including irinotecan, docetaxel, oxaliplatin, or oral 5-FU, are effective therapy options for advanced gastric cancer [61]. 

With so few reported cases, most which involved a single patient, it is difficult to correctly evaluate the response to postoperative chemotherapy of canine GC. Thus, it is extremely important to perform preliminary and thorough studies using in vitro models. The testing of anticancer drugs using cancer cell lines presents other advantages than just cytotoxicity evaluation tests, because it permits researchers to analyze the actions of drugs and of combinations of them, as well as to screen for resistance/sensitivity. The results of research in cancer cell lines are usually extrapolated to or tested in in vivo tumors [62], and may provide useful information on further steps in patient management. The outcome improvement depends on the development of a treatment strategy specific to GC and on adjuvant therapy schemes to improve life quality and as a modality of oncology management by reducing adverse treatment effects, pain, and discomfort associated with this disease [9].

## Figures and Tables

**Table 1:** Perioperative complications associated with canine gastric carcinoma surgical resection.

Surgery	Complications		Survival Time	References
Partial gastrectomy	Major intra- and postoperative complications	Spillage of gastric contents and septic peritonitis	1 day	[11]
		Inadvertent stab incision into small bowel and septic peritonitis	190 days	
	Major postoperative complications	Cardiopulmonary arrest	2 days	
		Septic peritonitis	2 days	
		Gastric stasis, pancreatitis, and cardiopulmonary arrest	15 days	
	Intraoperative complications	Minor hemorrhage	2 days	
			3 days	
			177 days	
	Minor postoperative complications	Hypertension	274 days	
		Hyporexia	132 days	
	Postoperative complications	Pericardial effusion	7 days	[12]
Partial gastrectomy and splenectomy	-	Disseminated intravascular coagulation and ventricular arrhythmias	2 days	
Partial distal gastrectomy and gastroduodenal anastomosis	Postoperative complications	Discomfort, vomiting, and diarrhea for 10–15 days	3 years	[14]
			4 years	
			30 days–20 months *	
			3 days	
			5 months	
Billroth I	Major postoperative complication	Pancreatitis	16 days	[11]
	Minor intraoperative complication	Hypertension	71 days	
	Postoperative complications	Ascending cholangiohepatitis and pancreatitis	49 days	
	Minor intraoperative complications	Major hemorrhage and 2nd-degree atrioventricular block	258 days	
	Major postoperative complications	Severe pancreatitis and intermittent hypoglycemia	183 days	
		Pulled out the gastrostomy tube		
	-	Persistent vomiting	3 days	[4]
	-	Vomiting and anorexia	6 weeks	
	-	Vomiting and anorexia	6 weeks	
	-	Vomiting and anorexia	10 months	
Billroth II	-	Vomiting and anorexia	4 weeks	
	-	Vomiting and anorexia	5 weeks	
Subtotal gastrectomy	Major intra- and postoperative complications	Septic peritonitis	13 days	[11]
	Minor intra- and postoperative complications	Hemorrhage and vomiting	93 days	
Total gastrectomy	-	Discomfort during and after eating	240 days	[25]
Pylorectomy and gastroduodenostomy	-		578 days **	[24]
			33 days ***	

* Six cases with survival time ranging from 30 days to 20 months; ** overall median survival time of 8 dogs that had complete excision of the disease; *** Overall median survival time of 5 dogs that had incomplete resection of the disease.

## Data Availability

Not applicable.

## Supplementary Materials

The following supporting information can be downloaded at: https://www.mdpi.com/article/10.3390/vetsci9080383/s1, Figure S1: Chemical structure of the drugs compounds used in canine gastric cancer treatment.
