# Supplementary material for: Canine Gastric Cancer: Current Treatment Approaches
Source: Vet Sci. 2022 Jul 26;9(8):383. doi: 10.3390/vetsci9080383 (PMC9394467; doi:10.3390/vetsci9080383)
Supplement: Supplementary file 1 [file vetsci-09-00383-s001.zip › vetsci-1746614-supplementary.pdf]

Supplementary Materials

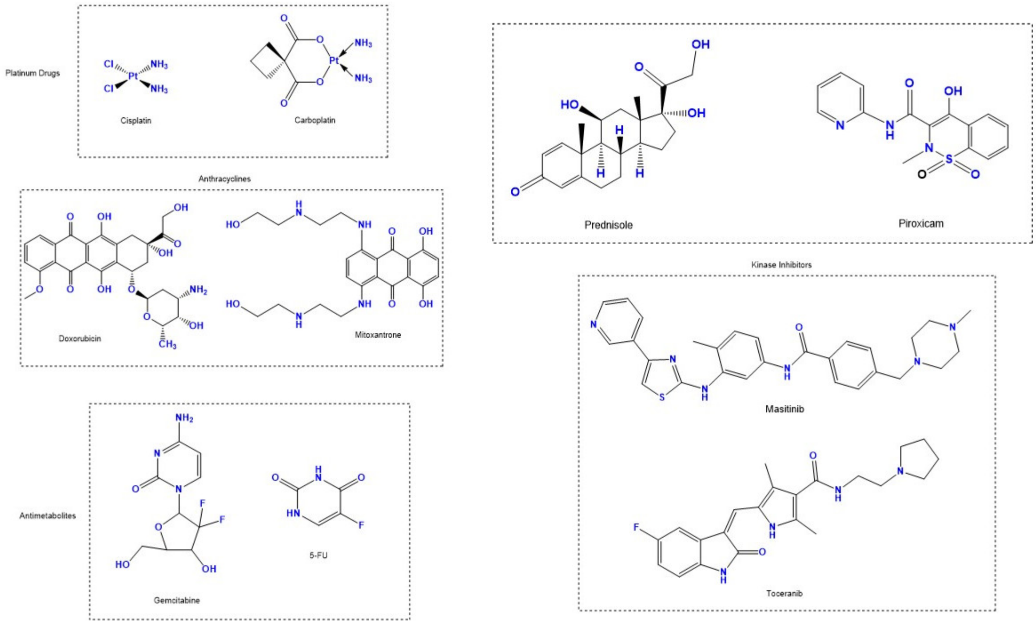

Figure S1. Chemical structure of the drugs coumpounds used in canine gastric cancer treatment.
